# A prospective, randomized comparison of ultrasonographic visualization of proximal intercostal block vs paravertebral block

**DOI:** 10.1186/s12871-020-0929-x

**Published:** 2020-01-09

**Authors:** Kamen Vlassakov, Avery Vafai, David Ende, Megan E. Patton, Sonia Kapoor, Atif Chowdhury, Alvaro Macias, Jose Zeballos, David R. Janfaza, Sujatha Pentakota, Kristin L. Schreiber

**Affiliations:** Department of Anesthesiology, Perioperative and Pain Medicine, Brigham and Women’s Hospital, 75 Francis Street, Boston, MA, 75 Francis St, Boston, MA 02115 USA

**Keywords:** Acute pain, Mastectomy, Paravertebral, Postoperative pain, Proximal intercostal, Truncal block, Ultrasound

## Abstract

**Background:**

Thoracic paravertebral blockade is an accepted anesthetic and analgesic technique for breast surgery. However, real-time ultrasound visualization of landmarks in the paravertebral space remains challenging. We aimed to compare ultrasound-image quality, performance times, and clinical outcomes between the traditional parasagittal ultrasound-guided paravertebral block and a modified approach, the ultrasound-guided proximal intercostal block.

**Methods:**

Women with breast cancer undergoing mastectomy (*n* = 20) were randomized to receive either paravertebral (*n* = 26) or proximal intercostal blocks (*n* = 32) under ultrasound-guidance with 2.5 mg/kg ropivacaine prior to surgery. Block ultrasound images before and after needle placement, and anesthetic injection videoclips were saved, and these images and vidoes independently rated by separate novice and expert reviewers for quality of visualization of bony elements, pleura, relevant ligament/membrane, needle, and injectate spread. Block performance times, postoperative pain scores, and opioid consumption were also recorded.

**Results:**

Composite visualization scores were superior for proximal intercostal compared to paravertebral nerve block, as rated by both expert (*p* = 0.008) and novice (*p* = 0.01) reviewers. Notably, both expert and novice rated pleural visualization superior for proximal intercostal nerve block, and expert additionally rated bony landmark and injectate spread visualization as superior for proximal intercostal block. Block performance times, needle depth, opioid consumption and postoperative pain scores were similar between groups.

**Conclusions:**

Proximal intercostal block yielded superior visualization of key anatomical landmarks, possibly offering technical advantages over traditional paravertebral nerve block.

**Trial registration:**

ClinicalTrials.gov, NCT02911168. Registred on the 22nd of September 2016.

## Background

Thoracic paravertebral blocks (PVBs) have been successfully used for analgesia in patients undergoing breast surgery, with considerable evidence that PVBs provide better postoperative pain control than systemic analgesia alone [1–5]. While complications are relatively rare [6], difficulties identifying the transverse process using the landmark-based technique may result in more frequent needle redirections, theoretically increasing the risk of pneumothorax (0.5%) [7].

### The opportunity and challenge of ultrasound guidance

Ultrasound (US) guidance has resulted in many proposed modifications [8, 9] to the PVB technique [10], promoting more widespread use [6, 11, 12]. However, real-time US visualization of these blocks remains technically challenging [13]. Goals of an optimal technique include: (1) continuous and simultaneous visualization of target and needle, (2) excellent pleural visualization to protect against pneumothorax, (3) sufficient distance from neuraxis to minimize bleeding risk, and (4) ease of performance for teaching trainees and practitioners.

### Paravertebral vs proximal intercostal spaces: anatomical considerations

The paravertebral space (PVS) is a wedge-shaped space lateral to the vertebral column where the spinal nerves emerge from the intervertebral foramina [14]. Its posterior boundary is formed by the superior costotransverse ligament (SCTL), which extends from the inferior aspect of each transverse process (TP) to the superior aspect of the rib below. The SCTL blends laterally with the internal intercostal membrane (IIM), which is the aponeurosis of the internal intercostal muscle and attaches medially to the upper and lower borders of the ribs. Thus, the lateral paravertebral space tapers and continues laterally into the proximal intercostal space (PICS), a transition that occurs near the costotransverse joint*.* The PICS is the medial-most segment of the intercostal space, bordered laterally by the angulus costae, cranially and caudally by adjacent ribs, posteriorly by the IIM and anteriorly by the endothoracic fascia/parietal pleura (PP) complex. Importantly, both the PVS and PICS contain the intercostal nerves, and the PVS also communicates inferiorly and superiorly with adjacent segmental PVSs, allowing a passageway for local anesthetic (LA) to spread to several adjacent segmental levels [15].

In the current study, we investigated the ability of a more lateral approach, the US-guided proximal intercostal block (PICB), to provide consistent real-time visualization of the wider, more superficial PICS just lateral to the transverse process and medial to angulus costae, and compared that to the visualization obtained with the more traditional PVB. We hypothesized that PICB would provide better visualization of pertinent structures (ribs, IIM, pleura, needle) than PVB (transverse process, SCTL, pleura, needle) as scored by blinded expert and novice reviewers of pre-insertion and pre-injection images and videoclips. We also assessed postoperative pain severity and opioid administration.

## Methods

### Patient and study characteristics

This prospective, randomized, controlled trial compared the parasagittal US-guided PVB with the US-guided PICB in patients undergoing unilateral or bilateral mastectomy with or without reconstruction. The study was approved by the Partners Institutional Review Board (IRB), Boston, MA, and registered with ClinicalTrials.gov, Identifier NCT02911168. Inclusion criteria included age 18–85 and ASA status I-III. Exclusion criteria included coagulopathy, allergy to local anesthetics, and patient refusal. Potential subjects were identified and approached in the anesthesia preoperative clinic. Written informed consent was obtained from all subjects, after which they were randomly assigned to one of two techniques (PVB or PICB), based on a previously determined computer-generated random list kept by one investigator, with all blocks performed on given patient following that type. The number of blocks each patient received was determined by the regional anesthesiologist, according to the procedure and (bi) laterality (1–4 separate block injections per patient). In total, 58 blocks were performed on 20 patients (26 PVB and 32 PICB). Patients and those assessing the outcomes and ultrasound images were blinded to the block received. Images and videos were collected, de-identified, and stored until rating.

### Perioperative

After placement of a peripheral intravenous catheter, standard ASA monitors, and supplemental oxygen, patients were placed in a supported seated position, and procedure and laterality were confirmed. Anxiolysis and analgesia were provided using midazolam and fentanyl, with continuous hemodynamic monitoring. All blocks were performed by regional staff anesthesiologists experienced in both techniques, or by advanced regional anesthesia fellows directly supervised by the former. Patients were fully blinded to their group assignment.

### Block technique

For both groups, high-frequency US imaging (Sonosite X-Porte, linear HFL50XP) was used in paramedian sagittal orientation. The first rib was identified, then ribs and transverse processes were visualized and counted cephalocaudally to identify the appropriate interspace(s). In both block groups, 1–2 thoracic interspaces between 1 and 6 were chosen, with the specific levels chosen to maximize coverage, each patient receiving 2.5 mg kg^− 1^ ropivacaine and 4 mg dexamethasone, with the concentration adjusted to provide clinically adequate volume per block level (0.5% for unilateral procedures, and 0.375% for bilateral procedures). At the end of the block, standard B- and M-mode lung scans were performed in the 3rd intercostal space to rule out pneumothorax.

### Technique for (traditional) US-guided PVB

After identifying the correct intercostal space, the US probe was used to scan medially over the adjacent transverse processes. The best possible parasagittal view of PVS, TPs, and pleura was then obtained and stored. A skin wheal was raised at the caudal border of the probe and a 21G, 10 cm echogenic SonoPlex block needle (Pajunk, GmbH, Geisingen, Germany) was inserted in-plane. The goal was to simultaneously visualize the needle and the injection target (immediately deep to the SCTL). Correct placement of the needle tip was confirmed visually by depression of the PP upon injection of LA, and by lack of retrograde spread of LA over the muscles or transverse processes. The LA was injected in 5 cc aliquots after negative aspiration of air, cerebrospinal fluid (CSF), or blood.

### Technique for US-guided PICB

After identifying the correct intercostal space, the US probe was used to scan medially over the adjacent TPs, then back laterally from the transverse processes tips to rest over the neck of the ribs and obtain the best possible view of ribs, PP, and IIM, and after skin wheel placement, block needle was inserted in-plane. The goal was to simultaneously visualize the needle and the injection target (immediately deep to the IIM, at the base of superior rib). Correct placement of the needle tip was confirmed visually by depression of the PP upon injection of LA, and by lack of retrograde spread of LA. The LA was injected in 5 cc aliquots after negative aspiration of air, cerebrospinal fluid (CSF), or blood.

### Block performance parameters

Imaging time was defined as the time interval between contact of the ultrasound probe with the patient and acquisition of a satisfactory US image. Needling time was defined as the time interval between the start of the skin wheal and the end of LA injection through the block needle. Number of needle passes, where the initial needle insertion was counted as “first pass”, and any subsequent needle advancement, preceded by a retraction of more than 2 cm, were counted. Distance of injection sites from the midline and depth of needle at skin upon injection were recorded.

### Primary and secondary outcomes

After all blocks were performed and images compiled, de-identified saved US images were independently rated by two reviewers, one expert (trained staff regional anesthesiologist with > 10 years of US-guided regional anesthesia experience), and one novice (staff anesthesiologist without specialized regional anesthesia training), who had not participated in the care of these patients. Each reviewer rated ability to visualize key structures in two still images and one video per block performed: (1) best image of block anatomy prior to needle placement (rated pleura, transverse process/rib, SCTL/IIM); (2) best image of block anatomy after needle placement, just before injection (rated pleura, transverse process/rib, SCTL/IIM, needle); (3) videoclip of block injection (rated LA spread visualization). Specified elements were graded on a 4-point Likert scale of visibility, as previously described [16], with 0 = not visible, 1 = hardly visible, 2 = well visible, 3 = very well visible. A composite score (0–15) was then calculated as the sum of the following 5 element scores: (1) average of pleura score before and after needle placement; (2) average of ligament score before and after needle placement; (3) average of bony element score before and after needle placement; (4) needle visualization score; and (5) injectate spread scores.

Secondary outcomes included numerical rating score of pain (0–10) at rest and with movement, 1 h after arrival in the post-anesthesia care unit (PACU) and 24 h after block placement, recorded nursing pain scores, as well as intraoperative opioid utilization, expressed as morphine mg equivalents (MMEs). Complications, including Horner’s syndrome, pneumothorax, local anesthetic systemic toxicity (LAST), were also recorded. Block success was verified postoperatively in the PACU by ascertaining presence of decreased sensation to pinprick in relevant dermatomal distribution.

### Statistical analysis

Patient characteristics were summarized using frequencies and percentages for categorical variables, and mean or median values with standard deviation or interquartile ranges (Q1-Q3) for continuous variables, according to normality of distribution. Testing for group differences was accomplished using Mann Whitney U test or independent samples t-test, as appropriate. All analyses were performed in SPSS 22. All statistical tests were two-tailed, with α = 0.05.

Sample size calculation was based on pilot estimations collected from US images obtained in the course of clinical care. Based on the scoring of a sample of images between 3 reviewers, we estimated mean scores of 9 and 12 for PVB and PICB blocks, respectively, and estimated a pooled standard deviation of 3. Using these estimated means and standard deviation and setting the power (1-β) to 0.9, and α to 0.05, we calculated that we would be able to detect a 3-point (20%) difference between groups with a sample of 20 blocks/group (40 total).

## Results

### Group comparison

All patients received their allocated randomized treatment, and images were captured and stored for each participant for analysis. There were no observed group differences in age, BMI, ASA status, unilateral vs. bilateral procedure, axillary node dissection, previous breast surgery, or presence of reconstruction (Table 1). Each patient received between 1 and 4 blocks of a single type, depending on the laterality and type of procedure, according to the clinical judgement of the attending anesthesiologist. Of those who had reconstruction, there was no difference in type (tissue expanders or DIEP flaps) between groups. Similarly, there was no significant difference in the overall type of anesthetic (total intravenous anesthetic general anesthesia vs. inhaled agent-based GA) or dose of intraoperative opioids (MME) used (Table 1). One patient in the PICB group had regional anesthesia (PICBs) as the primary anesthetic, with remaining patients receiving GA for the surgical procedure.

**Table 1:** Characteristics of Patients Randomized to PVB or PICB groups

	PVB (*n* = 10)	PICB (n = 10)	*P* value
Demographics			
Age	54 ± 11	47 ± 15	.259
BMI (Kg/m^2^)	27.1 ± 4.4	28.7 ± 6.1	.517
ASA	1 (0), 2 (8), 3 (2)	1 (1), 2 (8), 3 (1)	.513
Surgical Characteristics			
Surgical side(s)			
Unilateral	6	6	.675
Bilateral	4	4	
Axillary dissection	2	3	.500
Previous breast surgery	4	3	.871
Reconstruction	7	6	.500
Anesthetic Characteristics			
Type			
Volatile GA	7	7	.549
TIVA GA	3	2	
Sedation	0	1	
Intraoperative MME	53.5 ± 23.5	58.1 ± 19.6	.684

### Block characteristics

**Fig. 1:**
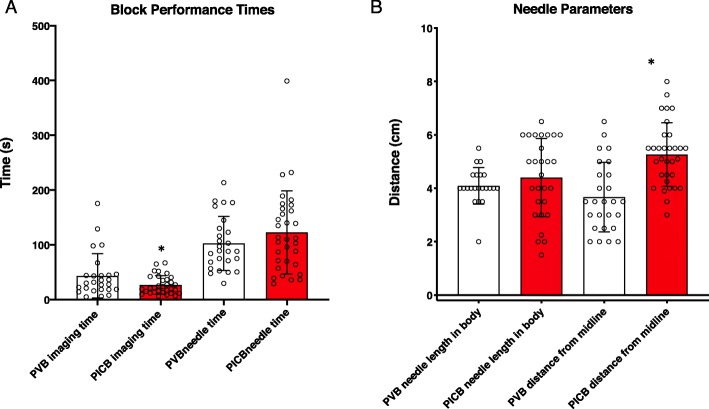
Block Characteristics (**a**) Imaging and needling times. Imaging, but not needling time was significantly lower for PICB than PVB (*p* = 0.245, *p* = 0.039, respectively). (**b**) Length of needle in body and distance from midline. Distance from midline was greater for PICB (*p* < 0.001), but no significance difference in length of needle in body(*p* = 0.363) was seen (Mann Whitney U-test). PICB: proximal intercostal block, PVB: paravertebral block

Imaging time was slightly lower for PICB compared to PVB (27 ± 17 vs. 43 ± 40 s) (Fig. 1a, Table 2), and PICB had a greater distance from midline (Fig. 1b, Table 2). Needling time, number of needle passes, and length of needle inside the body were similar between groups (Fig. 1a, Table 2). No instances of Horner’s syndrome, pneumothorax, or LAST occurred, and all patients reported decreased sensation to pinprick in relevant dermatomal distribution in PACU.

**Table 2:** Element and Composite Block Scores and Block Characteristics

Image Ratings		PVB (n = 26)	PICB (n = 32)	P value
Bony elements (TP/Rib)	Expert	2.08 ± .98	2.79 ± .37	.001
	Novice	2.14 ± .93	2.6 ± .52	.09
Pleura	Expert	1.9 ± 1.1	2.8 ± .42	.001
	Novice	1.9 ± .97	2.62 ± .59	.002
Ligament/IIM	Expert	1.67 ± 1.07	2.07 ± .89	.18
	Novice	1.33 ± .76	1.78 ± .82	.05
Needle	Expert	1.69 ± 1.12	2.21 ± 1.01	.07
	Novice	1.0 ± 1.06	1.59 ± 1.18	.06
Local Anesthetic Spread	Expert	2.23 ± .86	2.65 ± .72	.03
	Novice	1.73 ± .78	2.0 ± .89	.29
Composite Score	Expert	9.58 ± 4.22	12.48 ± 2.72	.008
	Novice	8.08 ± 3.28	10.57 ± 3.01	.01
Block Characteristics				
LA volume (cc)	14.2 ± 4.7		12.6 ± 3.2	.126
Imaging time (s)	43.3 ± 40.5		26.8 ± 16.8	.039
Needling time (s)	102.5 ± 49.1		122.7 ± 76.0	.245
Distance from midline (cm)	3.7 ± 1.3		5.3 ± 1.2	< 0.001
Length of needle in body (cm)	4.1 ± .70		4.4 ± 1.5	.363

### Ultrasound image quality

**Fig. 2:**
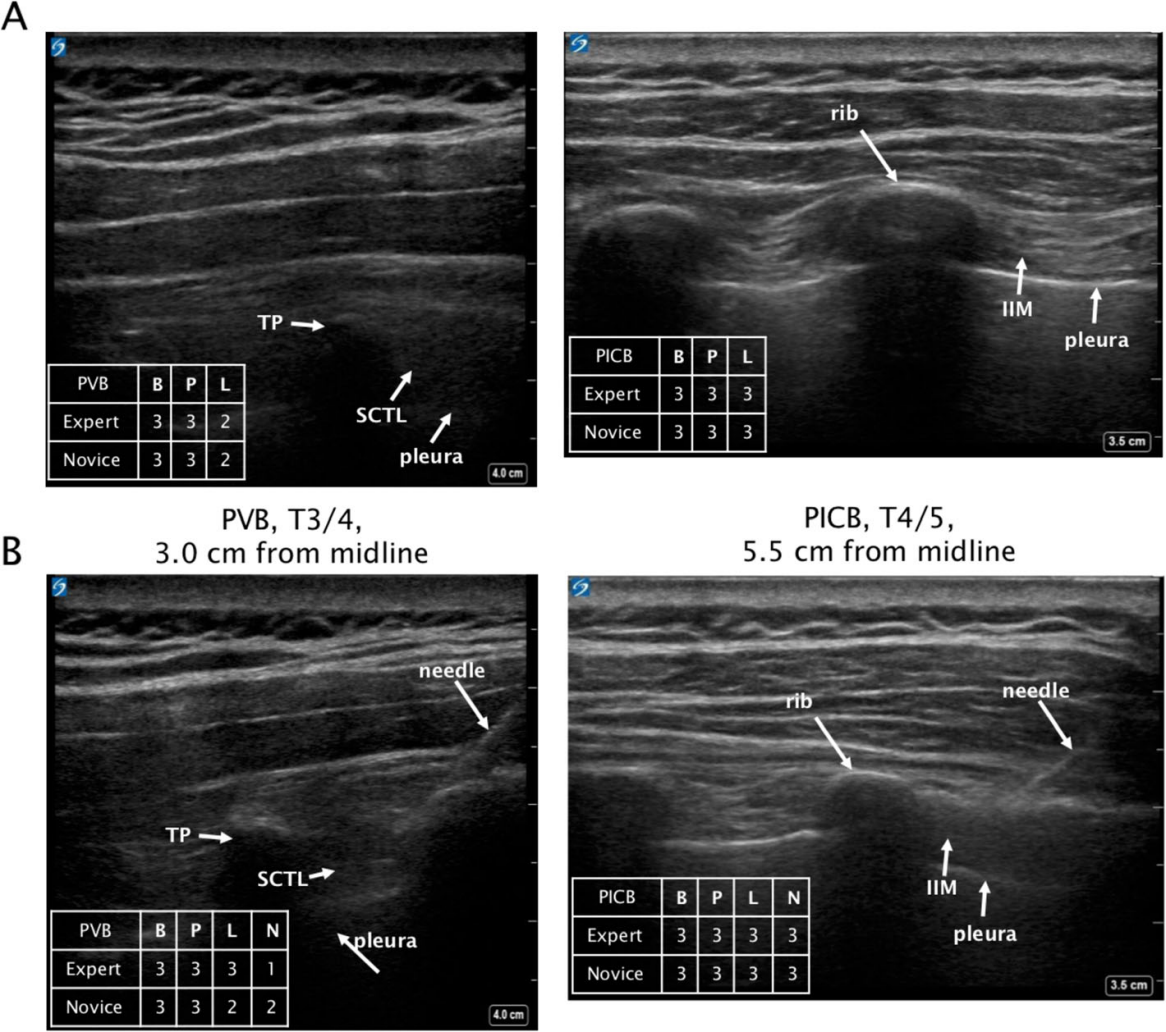
Ultrasound Images Representative highly rated block images before needle insertion for PVB (**a**) and PICB (**b**), and immediately before LA administration for PVB (**c**) and PICB (**d**). US visualization scores of expert and novice regarding quality of block elements are noted in tables on each image (B:bone, P:pleura, L:ligament/membrane, N:needle). PICB: proximal intercostal block, PVB: paravertebral block, LA: local anesthetic

**Fig. 3:**
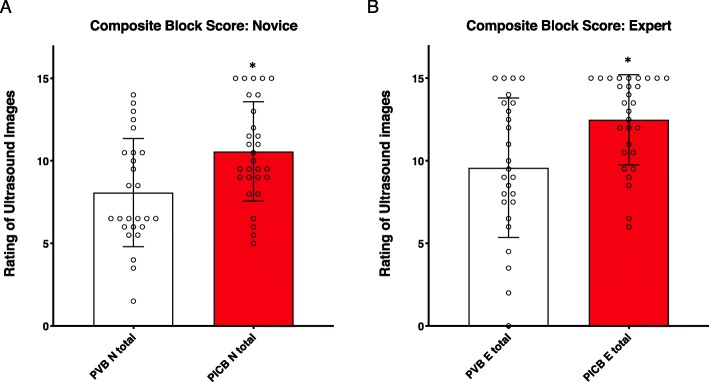
Block Visualization Scores Mean, standard deviation and distribution of block scores performed in patients randomized to PVB or PICB techniques. Scores were determined by anesthesiologists who were novice (**a**) or expert (**b**) at ultrasound imaging for regional anesthesia. Block scores were a sum of visibility of 5 elements, rated 0–3, including bony elements, pleura, ligament/membrane, needle, and local anesthetic spread, based on remote viewing of deidentified still images and short videoclip for each block. Both expert and novice reviewers rated PICB significantly higher (p = 0.008 expert, p = 0.01 novice, independent samples Mann Whitney U test)

US visualization quality of 5 block elements (bones, pleura, ligament, needle, LA spread; composite score = 0–15) was independently rated by blinded expert and novice reviewers. Still images before (Fig. 2a, Fig. 2b) and after (Fig. 2c, Fig. 2d) needle insertion were rated. For both expert and novice reviewers, composite visualization scores were higher in the PICB group (Fig. 3a, Fig. 3b, Table 2). Generally, expert block visualization ratings (Fig. 3b) were higher than novice ratings (Fig. 3a). In addition, expert-rated visualization scores of several individual elements (bone, pleura, and LA spread) were significantly higher for PICB, while the novice rated pleura and ligament visualization higher for PICB (Table 2).

### Pain ratings and opioid use

**Fig. 4:**
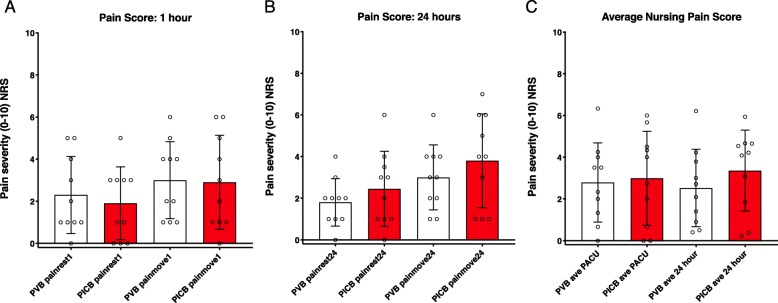
Pain Scores Pain in surgical area was rated at rest and with movement by patients on a 0–10 numerical scale, which showed no significant difference in scores between groups at (**a**) 1 and (**b**) 24 h after surgery. Inpatient pain scores collected by nurses over the first 24 h were also extracted from the electronic medical record and averaged for each patient (**c**), revealing no difference in average clinically reported pain over this period between groups *p* = .597 for 1 h, *p* = .436 for 24 h, *p* = .739 for average 24 h nursing score. (independent samples Mann Whitney U test)

Patients in PVB and PICB groups rated pain at rest and with movement 1 h after PACU arrival (Fig. 4a) and 24 h after block placement (Fig. 4b) similarly. Additionally, pain scores recorded in the electronic medical record and averaged for PACU and post-PACU-24 h time periods were also not different between groups (Fig. 4c). There was no significant difference observed in intraoperative opioid administration between groups (Table 1), and no significant complications were observed in either group.

## Discussion

In this study, we compared the quality of ultrasonographic visualization of relevant landmarks, block needle and injectate spread obtained with two truncal regional anesthesia techniques: the traditional parasagittal US-guided paravertebral block (PVB), and an US-guided proximal intercostal block (PICB) technique. PICB yielded superior overall visualization scores, as well as superior pleura visualization ratings, rated by both expert and novice US image reviewers. Clinically, better visualization not only has the potential to improve block success and safety, but also teaching, learning, and practitioner confidence.

Intercostal nerve block has been used for analgesia after mastectomy, using a landmark based approach [17]. With the addition of US guidance, our group has clinically employed the PICB technique since 2013 as an alternative to PVB in a variety of cases: mastectomy, thoracic, cardiac, and abdominal surgery, as well as rib fracture, where it has provided effective analgesia, both as single injections and continuous blocks (unpublished observations). The current randomized study formally compared the quality of US visualization between these two approaches in a pragmatic setting in mastectomy patients.

Individual elements were better discerned by the expert than the novice reviewer for both types of blocks, likely due to a greater familiarity with reading US images, although both reviewers rated composite visualization for PICB superior to PVB. Individual block elements that were scored higher for PICB included bony elements, pleura, and LA spread (expert) and pleura and ligament/membrane (novice). The finding that pleura visualization was clearly superior for both expert and novice reviewers is significant, since pneumothorax is arguably the most feared PVB complication (~ 0.5%) [6]. Within the context of training residents and fellows in regional anesthesia, visualization of pleura, which varies substantially between patients, remains independently and critically important for accurate and safe needle guidance [18].

Given the relatively deeper location of TP to ribs, the lack of difference in length of needle insertion (i.e. cm marking at skin) between PVB and PICB is, at first glance, surprising. This, together with the larger variations in the length of needle insertion in the PICB group (mean ± standard deviation: 4.4 ± 1.5 cm) than the PVB group (4.1 ± 0.7 cm), could be explained by a wider range of permissible needle trajectory angles for PICB. The spaces between the adjacent ribs are usually wider than the spaces between the TPs, thus allowing shallower needle to US-probe angles, which might be responsible for the trend towards better visualization of the block needle, but also result in longer skin-target distances.

PVB was initially described using landmark technique, with needle insertion point at a fixed, anatomically-based distance of 2.5–3.0 cm lateral from the spinous processes [13, 19]. After the introduction of US guidance, PVB has rapidly evolved, with many modified techniques described and adopted clinically, all aiming at assuring easier and safer access to the PVS [11, 13, 20]. Among those, the paramedian sagittal approach most closely resembles the traditional landmark technique, but uses individual sonoanatomy rather than a predetermined distance from the midline to guide needle placement. Interestingly, using this method, our PVB needle entry point (3.7 ± 1.3 cm) was somewhat more lateral than the classically described 2.5 cm, but still medial to the PICB (5.3 ± 1.2 cm).

A more lateral block technique may offer several important advantages: (1) reduced risk of inadvertent neuraxial block and hematoma, due to greater distance from spinal canal, (2) ability to orient US probe at an angle more perpendicular to the pleura, and (3) less steep needle angle trajectory, as PICS is typically shallower and wider than PVS. Previous more lateral approaches to the PVS have been described, using either landmark- or US-guidance [11, 12], but in these cases the target injection site was the PVS itself, rather than the PICS. The lateral to medial transverse or oblique needle trajectory described in these techniques, however, is somewhat concerning for a potentially higher incidence of inadvertent epidural injection/catheter placement [15, 21].

Physical needle entry into the PVS may not be necessary to obtain effective multi-level analgesia if sufficient volume is injected into an adjacent contiguous/communicating space [20, 22]. Other methods have attempted to creatively approach truncal block, using easy to master US-guided block techniques, such as the retrolaminar [23] and erector spinae plane blocks [24]. An important consideration with any indirect approach is that LA spread may be less consistent and more prone to anatomical variations the farther the injection point is from the target, although this may be overcome by relatively higher volume injection [25, 26]. Earlier anatomical study of *lateral* intercostal injections (at the costal angle, much farther lateral to the PICB) failed to produce extensive spread beyond the level of injection, even with larger volumes (20 ml), as measured by CT and cutaneous sensory testing [27]. In contrast, Nunn et al. [28] demonstrated significant spread in all directions: medially (PVS), laterally (intercostal space), cephalad, and caudad (adjacent intercostal space). Nunn’s results are in agreement with our own preliminary anatomical findings for PICB injection in cadavers, which also result in substantial cephalo-caudal spread of LA along the endothoracic fascia and via the PVS [29]. Interestingly, Naja and colleagues suggest that injections anterior to the endothoracic fascia result in more consistent paravertebral spread than those posterior to this structure [30].

There are several limitations to this study. First, although the number of blocks was adequate to power the primary outcome (US visualization quality), the number of subjects was relatively small, likely providing inadequate power to detect differences in the secondary endpoints of pain and opioid use between the groups. Second, the blinding of the expert reviewer may arguably be compromised, as images provide clues to block-specific sonoanatomy. Third, although we compared expert and novice assessment of US image quality, we did not test the relative ability of novices, more advanced learners under supervision, and experts, in capturing and using their own US-images to guide block performance. Last, although we did not observe any complications, the number of subjects in this study was insufficient to draw any conclusions about the relative safety of these block techniques on a larger scale. The absence of complications in either group is not surprising given the small number of blocks (58), and their performance by experienced clinicians. Given the previously reported higher complication rates with trainees [22, 31, 32], the relative importance of the PICB visualization advantages may be more relevant in a training context.

## Conclusions

In conclusion, this study shows improved US visualization scores with the PICB technique over the traditional PVB, as rated independently by expert and novice reviewers. In the context of an academic teaching hospital, better sonoanatomy visualization with PICB has the potential to improve block success and safety and facilitate teaching, learning, and practitioner confidence, while providing comparable clinical results to the traditional PVB.

## Data Availability

The datasets generated during the current study are not publicly available due to HIPAA restrictions but are available from the corresponding author on reasonable request.

## Abbreviations

BMI: Body mass index
CSF: Cerebrospinal fluid
DIEP: Deep inferior epigastric perforator
EF: Endothoracic fascia
GA: General anesthesia
IIM: Internal intercostal membrane
LA: Local anesthetic
LAST: Local anesthetic systemic toxicity
MME: Morphine mg equivalent
PACU: Post-anesthesia care unit
PICB: Proximal intercostal block
PICS: Proximal intercostal space
PP: Parietal pleura
PVB: Paravertebral blockade
PVS: Paravertebral space
SCTL: Superior costotransverse ligament
TP: Transverse process
US: Ultrasound
